# Codeless Development of a Customized SMILE Nomogram Using a Large Language Model: A Practical Framework for Clinicians

**DOI:** 10.1155/joph/9930116

**Published:** 2025-07-15

**Authors:** Hye Won Jun, Sun Young Ryu, Tae Keun Yoo

**Affiliations:** ^1^Department of Ophthalmology, Hangil Eye Hospital, Incheon, Republic of Korea; ^2^Department of Refractive Surgery, B&VIIT Eye Center, Seoul, Republic of Korea

**Keywords:** calculator, ChatGPT-4, no-code development, nomogram, SMILE

## Abstract

**Purpose:** To evaluate the feasibility of using ChatGPT-4, a large language model (LLM), to develop a customized nomogram calculator for small-incision lenticule extraction (SMILE) surgery based on institution-specific data, without requiring any coding expertise. Customized nomograms are essential due to variations in surgical practices, patient populations, and diagnostic equipment across vision correction centers.

**Methods:** A retrospective analysis of consecutive patients was performed on data of 1268 eyes that underwent SMILE. Preoperative measurements and postoperative refractive errors at 6 months were collected and analyzed. The entire dataset was divided into a training set and validation set at a ratio of 3:1. After data anonymization, ChatGPT-4 was instructed to perform a linear regression analysis to predict postoperative refractive errors using preoperative data. Subsequently, we instructed ChatGPT-4 to generate HTML code for a webpage-based nomogram calculator that inputs preoperative data and calculates surgical parameters using the derived formulas. The results of the regression analysis performed using ChatGPT-4 were compared with those obtained using two conventional statistical software programs, R and SPSS.

**Results:** ChatGPT-4 successfully performed SMILE nomogram regression analysis. The predicted SMILE parameters were not significantly different from those obtained using the statistical software. The nomogram showed a higher predictive ability for postoperative refractive error than the simple empirical nomogram (*p* < 0.001). We successfully created a webpage-based calculator using ChatGPT-4 through multiple prompt instructions without coding.

**Conclusion:** ChatGPT-4 not only provides a statistical model for SMILE nomograms but also creates a calculator for user convenience. Clinicians can easily build their own nomogram calculators using only the collected data without coding. The advanced LLM will allow clinicians to conveniently create customized nomogram tools.

## 1. Introduction

As the prevalence of myopia increases worldwide, the number of laser vision correction surgeries is also rising [1]. In vision correction surgeries, the development of a nomogram for accurate clinical outcome prediction is very important to correct refractive errors as desired [2]. Nomogram refers to a method of calculating values input to the actual laser machine for surgery [3]. Through a well-established nomogram, good surgical results can be obtained by numerically analyzing the patients' clinical results and values that are entered into the actual laser machine. Even in small-incision lenticule extraction (SMILE), the design of a nomogram is essential to avoid over- and under-correction [4].

Inter-institutional differences in manifest refraction (MR) protocols and variability in diagnostic equipment can lead to inconsistencies in ocular measurements. These factors complicate the development of a universally applicable nomogram. Given the inherent variability in surgical techniques, patient demographics, and instrumentation across centers, nomograms for refractive surgery are highly institution-specific. Consequently, a standardized, one-size-fits-all nomogram often fails to achieve optimal outcomes. To enhance accuracy and surgical predictability, it is essential for each vision correction center to construct a customized nomogram tailored to its clinical environment and patient population. However, the process of performing statistical analyses and implementing these formulas into usable software tools remains labor-intensive and technically challenging.

**Figure 1:**
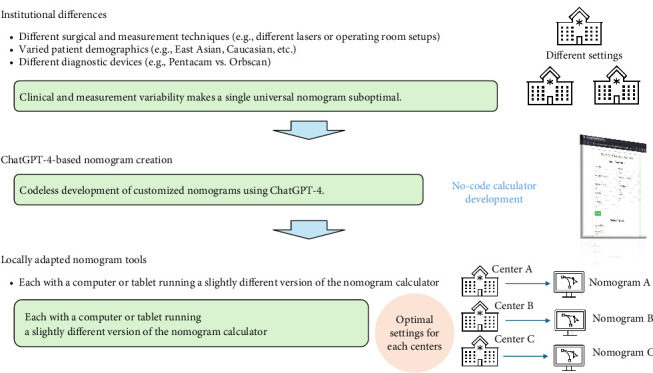
Importance of local customization in nomogram development using LLM. Due to institutional differences in surgical techniques, patient populations, and diagnostic devices, a single universal nomogram is often suboptimal. This study proposes a codeless approach using ChatGPT-4, where each center can independently develop customized nomograms based on their own datasets. ChatGPT-4 performs linear regression and generates web-based calculator code through prompt instructions, enabling clinicians to deploy institution-specific nomogram calculators (Nomograms A, B, and C) optimized for local settings.

With the rapid development of artificial intelligence (AI), ophthalmological institutions can easily and conveniently develop nomograms based on their data. Recently, several tools that can perform numerical analyses, such as regression analysis without coding, have been introduced [5]. However, they require knowledge of basic data analysis. The recent emergence of ChatGPT, an advanced large language model (LLM), has facilitated no-coding–based inferencing, analysis, and programming [6]. ChatGPT-4 is trained on data related to medical fields and is able to form relevant connections to interpret and calculate various formulas [7, 8]. In epidemiological studies, statistical calculations using ChatGPT-4 have been reported to provide accurate results [9]. This allows automated software development without coding using appropriate prompts; therefore, many developers are already applying it [10]. In particular, the concept of vibe coding has enabled non-experts to develop software using LLMs through natural language prompts [11]. In the field of refractive surgery, the application of LLMs is also expanding, offering new possibilities for data-driven personalization and clinical decision support [12]. Our approach using ChatGPT-4 enables this by allowing clinicians to generate center-specific nomogram formulas and calculators without coding. This framework can be replicated by other institutions, facilitating widespread, individualized implementation (Figure 1).

In this study, we developed a nomogram based on pre- and postoperative data of SMILE vision correction surgery using ChatGPT-4 and showed that it could be used to create an easily usable calculator. This method can enable individual institutions to improve surgical outcomes by simply developing customized nomograms using the prompts given in our study.

## 2. Materials and Methods

### 2.1. Dataset

The dataset used in this study was based on a retrospective chart review of consecutive patients. This study included patients who underwent SMILE at the B&VIIT Eye Center between 2020 and 2021 and were followed up for 6 months. Patients who did not complete the pre- and postoperative examinations or had incomplete medical chart data were excluded. The authors used fully de-identified data for analysis. The Institutional Review Board (IRB) of the Korean National Institute for Bioethics Policy approved the study's retrospective data collection protocol. The requirement for informed consent was waived due to the retrospective nature of the study by the IRB. All the research procedures were conducted in accordance with the principles of the Declaration of Helsinki.

The dataset was randomly divided into training and validation sets in a 3:1 ratio. To maintain independence between subjects, randomization was performed at the patient level so that both eyes of a given patient were assigned to the same set. The data partitioning process was subsequently rechecked and confirmed to ensure that no subject overlap occurred between the two sets. Nomogram formulas were developed using the training sets. The formulas were evaluated using a validation set.

### 2.2. Data Collection

All patients underwent preoperative evaluations, including MR, Pentacam (Oculus GmbH, Wetzlar, Germany), and Keratograph 5M (Oculus GmbH, Wetzlar, Germany). All the SMILE procedures were performed by 10-years expert surgeons with a standard manner. A VISUMAX 500 fs laser platform (Carl Zeiss Meditec AG, Jena, Germany) was used to cut the corneal lenticules. The incision cut and size were 145° and 2.0 mm, respectively. The thickness of the cap was 120 μm. The surfaces of the lenticules were dissected using a spatula, and the lenticules were extracted using forceps. The postoperative evaluation included MR 6 months after surgery.

Preoperative data collection included manifest refraction values for sphere (MR_sph) and cylinder (MR_cyl), central corneal thickness (CCT), pupil size under photopic conditions, and corneal biometric parameters such as mean anterior keratometry (K_mean) and corneal astigmatism (K_astig), which were obtained using the Pentacam system. The white-to-white (WTW) corneal diameter was measured using the Keratograph 5M. Although primarily designed for ocular surface evaluation, the Keratograph 5M has been shown to provide reliable and reproducible WTW measurements. At the center, this device is preferred over alternatives such as the Pentacam or anterior segment OCT, aligning with the center's routine preoperative assessment protocols. The axis of manifest astigmatism (MR_axis) was binarized between 45° and 135° and was coded as 1 for against-the-rule astigmatism and 0 for with-the-rule astigmatism. This is based on previous research in which the nomogram had to be different depending on the direction of astigmatism [13]. The surgical inputs into VISUMAX 500 including sphere, cylinder, and optical zone (OZ) diameters were also collected. The final structure of the dataset is shown in Supporting Information 1.

### 2.3. Nomogram Development

ChatGPT-4 was employed as a decision support tool to assist with variable selection and the structuring of regression formulas. Direct computation of the nomogram using patient-level datasets was not conducted within ChatGPT itself and was considered beyond the scope of this study. Similar to the method given in a previous study, we selected the eyes with ideal 6-month postoperative refractive outcomes (sphere within ±0.50 D and cylinder within ±0.50 D) to develop a nomogram. The collected preoperative features, including MR_sph, MR_cyl, MR_axis, CCT, pupil size, K_mean, K_astig, and WTW, were used as input factors to develop the nomogram. Using these input values, we created a formula via linear regression to predict the sphere (Parameter_SPH), cylinder (Parameter_CYL), and OZ diameter (Parameter_OZ) input parameters for the VISUMAX 500. Linear regression was selected for this study due to its interpretability and clinical transparency, which are critical in surgical planning and nomogram development. The dependent variables (Parameter_SPH, Parameter_CYL, and Parameter_OZ) were continuous and no significant violations of linearity, homoscedasticity, or multicollinearity were observed in preliminary diagnostics. ChatGPT-4 was instructed to find the formula using linear regression with feature selection. ChatGPT-4 was used to assist with regression model development and variable selection. Specifically, the GPT-4-turbo model, as deployed by OpenAI on the ChatGPT platform (version dated November 2023), was accessed via chat.openai.com between March and July 2024. To verify its accuracy, linear regression with stepwise backward elimination was performed using R software (Version 4.4.0) and SPSS (Version 23.0; IBM, Chicago, Illinois, USA). Machine learning methods were excluded because they cannot be reliably developed or validated using ChatGPT or other no-code tools, which were central to the study's design framework.

### 2.4. Software Summary

**Figure 2:**
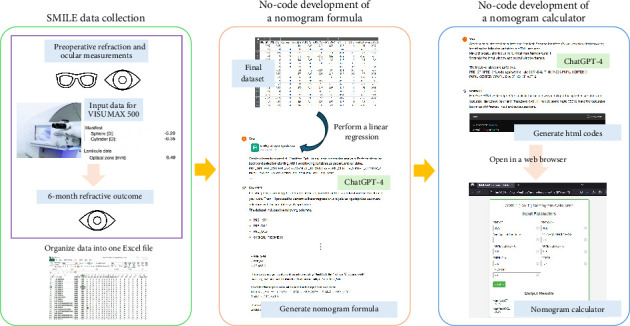
Workflow for no-code development of a customized SMILE nomogram using ChatGPT-4. The process consists of three main steps: (1) SMILE data collection, where preoperative and postoperative clinical measurements are collected and organized into an Excel dataset; (2) no-code development of a nomogram formula, where ChatGPT-4 is prompted to perform linear regression with feature selection to generate a predictive model for surgical input parameters; and (3) no-code development of a nomogram calculator, where ChatGPT-4 generates HTML code for a web-based calculator based on the derived formulas, enabling clinicians to input preoperative values and obtain customized VISUMAX 500 parameters without coding.

As shown in Figure 2, we focused on developing a customized nomogram calculator using ChatGPT-4, which is a prompt-based AI with strong reasoning. ChatGPT-4 does not require a special programming language for the development. In addition, the prompts do not require strict formatting as the LLM is able to interpret human language. Users can use the chat window to send data, derive formulas, and develop software using ChatGPT-4 with a web browser. If developed in Hypertext Markup Language (HTML), which can run on any web browser, creation of a nomogram calculator is possible that can be executed using different operating systems. ChatGPT-4 is trained using publicly available data (https://openai.com/research/gpt-4). All prompts written by the authors and codes generated in this study belong to this paper.

Table 1 shows the prompts used to perform linear regression and build a nomogram calculator. The prepared Excel data file was displayed in the first dialog window. Here, the input variables were sequentially instructed to obtain the sphere, cylinder, and OZ diameter input parameters for the VISUMAX 500 formula through linear regression analysis accompanied by feature selection. Since ChatGPT-4 understands the context of a conversation, all subsequent calculations are possible using only one data input. After establishing all the formulas, we instructed ChatGPT-4 to create HTML codes for a nomogram calculator. The calculator and the arrangement of the inputs and outputs were specifically explained.

### 2.5. Other Methods

The formula developed using ChatGPT-4 was compared against two additional regression approaches and a previously reported simple empirical nomogram. The simple nomogram was based on a fixed adjustment of preoperative MR values, defined as [nomogram parameters] = [preoperative MR values] − 0.25– 0.11 ∗ [preoperative MR values] [14]. In addition, linear regression models were independently developed using two conventional methods for comparison. The first was an R-based backward stepwise feature elimination approach, implemented using the “stepAIC ()” function from the MASS package (Supporting Information 2). The second was a backward stepwise regression performed in SPSS, applying a statistical threshold of *p* < 0.01 for variable inclusion and retention. Both models were trained on the same dataset and optimized to predict three target parameters: Parameter_SPH, Parameter_CYL, and Parameter_OZ. The resulting formulas were evaluated on a separate validation set to assess their performance relative to the ChatGPT-4-derived model and the simple empirical nomogram.

### 2.6. Statistical Analysis

In the validation set, refractive prediction errors for each nomogram were calculated and compared based on the MR measured 6 months after surgery. The change in MR between the preoperative and 6-month postoperative visits was assumed to reflect the surgical correction. Refractive prediction errors were determined by comparing the achieved correction with the correction amounts estimated by each formula, using the actual sphere and cylinder parameters applied by the VISUMAX 500 platform.

Each variable was compared using a paired *t*-test with Bonferroni correction for multiple comparisons. A paired *t*-test was chosen because the same eyes were evaluated across all nomogram models, allowing direct within-subject comparison of prediction errors. A Bonferroni-corrected *p* value of less than 0.05 was considered statistically significant.

## 3. Results

### 3.1. Baseline Information

This study included data of 1268 eyes from 752 patients who underwent SMILE. At the patient level, 950 eyes (75%) were assigned to the training set and 318 eyes (25%) to the test set.  Table 2 presents the ocular parameters used in this study. There was no statistically significant difference between the training and test sets for all other factors except astigmatism (cylinder) 6 months after surgery (*p*=0.001). The preoperative MR sphere values of the training and test sets were −3.64 ± 1.28 D and −3.78 ± 1.32, respectively, and the MR cylinder values were −0.84 ± 0.65 D and −0.80 ± 0.59 D, respectively. In both sets, the against-the-rule astigmatism direction was observed in 9.4% of the patients.

### 3.2. Development of SMILE Nomogram

**Figure 3:**
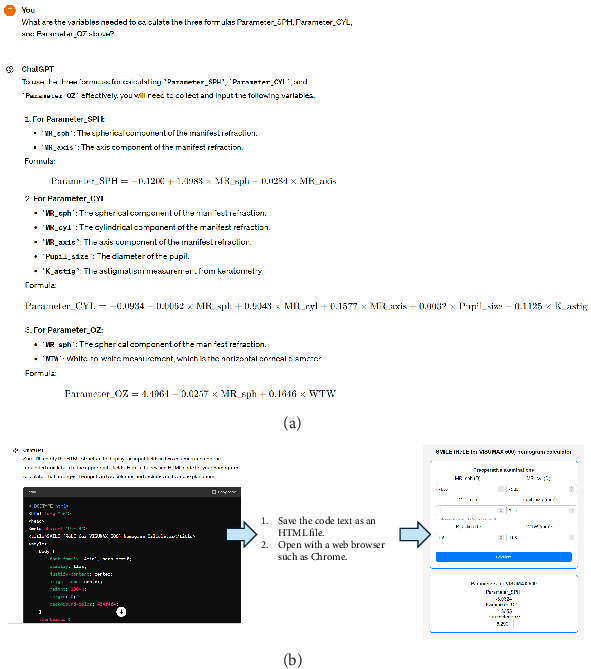
Operation of ChatGPT-4 to build a nomogram calculator. (a) Screenshot of a dialog in ChatGPT-4 to develop the formulas. (b) The code generation and calculator built by ChatGPT-4. The axis of manifest astigmatism (MR_axis) was binarized: between 45° and 135° and was coded as 1 for against-the-rule astigmatism and 0 for with-the-rule astigmatism.

During the development process using ChatGPT-4, the researchers did not perform any coding or mathematical calculations. ChatGPT-4 successfully recognized the training data in an Excel file and summarized the means and variances of the variables. As instructed via prompts, ChatGPT-4 performed a linear regression analysis with feature selection to build the formulas for the sphere, cylinder, and OZ diameter input parameters for the VISUMAX 500 (Figure 3(a)). Without the use of specific commands, intercorrelations between variables were analyzed for feature selection, related factors were extracted, and variables for regression analysis were selected (Supporting Information 3).

After establishing all the formulas, we immediately ordered the production of a calculator based on the HTML language (Figure 3(b)). ChatGPT-4 automatically generated code that could be run on a web browser to create a nomogram calculator based on conversation history without the requirement of specifying detailed inputs, outputs, or formulas. As shown in Table 1, detailed design instructions were provided to create a convenient and user-friendly calculator. The generated codes are shown in Supporting Information 4 (the calculator is available on https://taekeuntoo.github.io/MySmileCalc/). The researchers ran the saved HTML code file in all web browsers–Chrome, Firefox, and Edge–and confirmed that the nomogram calculator worked well without errors using the formula set.

### 3.3. Validation of SMILE Nomogram

**Figure 4:**
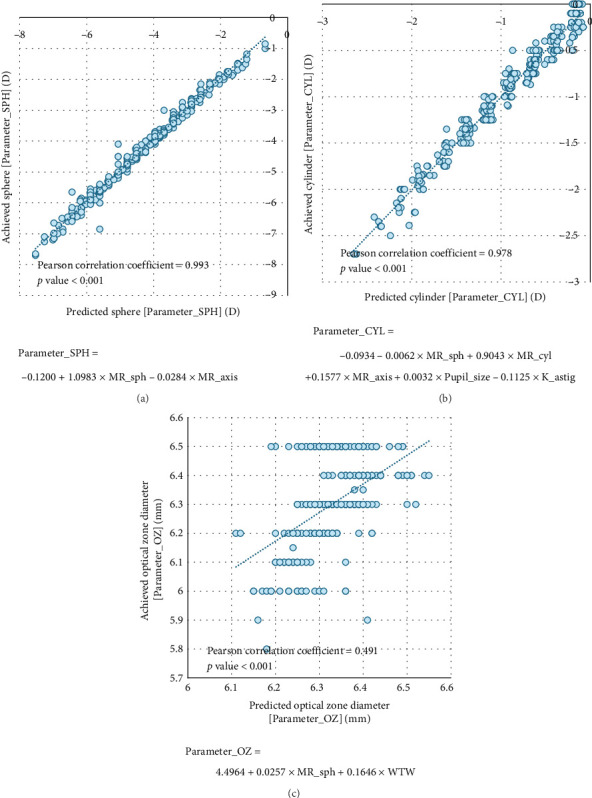
Comparison of the achieved parameters and results predicted by ChatGPT-4's formulas in the test dataset. VISUMAX 500 input parameters of (a) sphere, (b) cylinder, and (c) optical zone diameter.

Figure 4 shows the distribution of the nomogram formulas developed in ChatGPT-4 and the parameters obtained from the test dataset. The sphere, cylinder, and OZ diameter parameters showed Pearson correlation coefficients of 0.993 (*p* < 0.001), 0.979 (*p* < 0.001), and 0.491 (*p* < 0.001) between the achieved and calculated values, respectively.

To evaluate the accuracy and consistency of the nomogram formulas generated by ChatGPT-4, we compared them with regression models developed using SPSS and R on the same training dataset. The results of the linear regression with backward elimination performed in SPSS are presented in Table 3. This result is slightly different from the calculation result of ChatGPT-4 in Table 2. ChatGPT-4 only uses MR_sph and MR_axis to calculate the input sphere, while SPSS additionally requires MR_cyl for calculation. Additionally, ChatGPT-4 used MR_sph, MR_cyl, MR_axis, Pupil_size, and K_astig to calculate the input cylinder, while SPSS did not use Pupil_size. Since the variable selection method was different from that of ChatGPT-4, the final selected variables and coefficients were different. In the formulas built using SPSS, the CCT, which was not used in ChatGPT-4, was additionally used, and more factors were included in each formula. Similarly, R-based models generated using backward stepwise elimination (Supporting Information 5) included MR_sph, MR_cyl, and K_astig for sphere prediction; MR_sph, MR_cyl, MR_axis, and K_astig for cylinder prediction; and MR_sph, WTW, and CCT for OZ prediction. Despite minor variations, the overall structure of the regression models was consistent across platforms.

Table 4 presents the prediction error metrics for each nomogram model across sphere, cylinder, and spherical equivalent outcomes. The models include ChatGPT-4, R, SPSS, and the Simple nomogram. For sphere prediction, the SPSS model showed the lowest mean absolute error (0.3077 D) and root mean square error (0.3782 D), indicating slightly better performance than the other models. In cylinder prediction, the Simple nomogram had the lowest errors, with a mean absolute error of 0.1817 D and a root mean square error of 0.2388 D, although this model showed limited accuracy in other components. For spherical equivalent prediction, the SPSS model again showed the lowest root mean square error (0.3282 D), while all three regression models performed similarly. The Simple nomogram showed the highest errors in both sphere and spherical equivalent prediction, with values over 0.43 D for mean absolute error and over 0.50 D for root mean square error, suggesting reduced accuracy compared to the regression-based methods.

**Figure 5:**
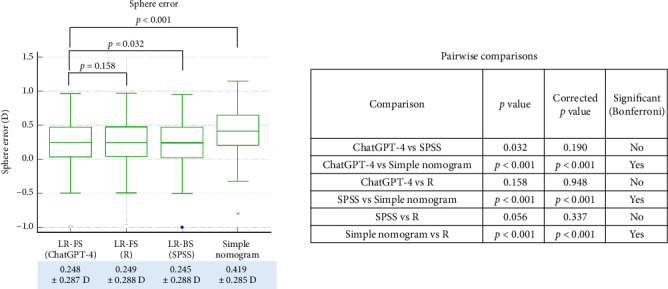
Comparison of spherical prediction errors, calculated as the difference between the achieved postoperative correction and the correction predicted by each nomogram model in the test dataset. LR-FS: linear regression with feature selection performed by ChatGPT-4 or R; LR-BS: linear regression with stepwise backward elimination performed using SPSS.

**Figure 6:**
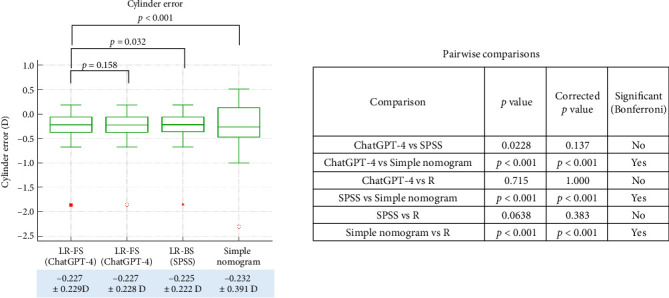
Comparison of cylindrical prediction errors, calculated as the difference between the achieved postoperative correction and the correction predicted by each nomogram model in the test dataset. LR-FS: linear regression with feature selection performed by ChatGPT-4 or R; LR-BS: linear regression with stepwise backward elimination performed using SPSS.

**Figure 7:**
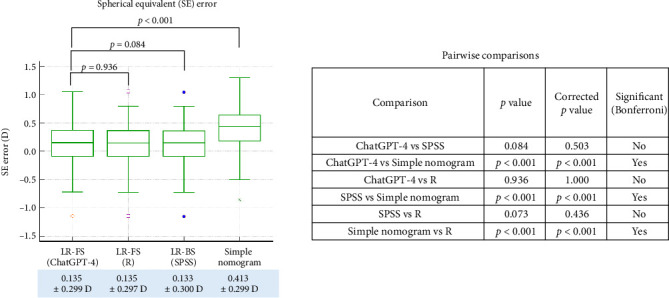
Comparison of spherical equivalent prediction errors, calculated as the difference between the achieved postoperative correction and the correction predicted by each nomogram model in the test dataset. LR-FS: linear regression with feature selection performed by ChatGPT-4 or R; LR-BS: linear regression with stepwise backward elimination performed using SPSS.

Finally, we evaluated the prediction accuracy of each nomogram by comparing the achieved postoperative refractive outcomes to the correction values estimated from each formula using the 6-month MR data. In terms of sphere prediction error (Figure 5), the ChatGPT-4-based model (linear regression with feature selection) showed a mean error of −0.248 ± 0.287 D, which was not significantly different from the R-based regression model (−0.249 ± 0.288 D, *p*=0.158, corrected *p*=0.948). Likewise, no significant difference was observed between ChatGPT-4 and SPSS-based regression (−0.245 ± 0.288 D, *p*=0.032, corrected *p*=0.190). However, the sphere error from the simple empirical nomogram (−0.419 ± 0.285 D) was significantly higher than all three regression models (all corrected *p* < 0.001). For cylinder prediction error (Figure 6), the ChatGPT-4-based model (−0.227 ± 0.229 D) again showed no significant difference compared to the R-based model (−0.227 ± 0.228 D, *p*=0.715, corrected *p*=1.000) or the SPSS-based model (−0.225 ± 0.222 D, *p*=0.0228, corrected *p*=0.137). The simple nomogram, which yielded a larger error of −0.232 ± 0.391 D, was significantly less accurate than all three regression-based models (all corrected *p* < 0.001). With regard to spherical equivalent error (Figure 7), ChatGPT-4 (−0.135 ± 0.299 D) and the R-based model (−0.135 ± 0.297 D) again produced highly similar results (*p*=0.936, corrected *p*=1.000). No significant difference was found between ChatGPT-4 and SPSS (−0.133 ± 0.300 D, *p*=0.084, corrected *p*=0.503). However, the simple nomogram exhibited a substantially larger SE error (−0.413 ± 0.299 D), which was significantly greater than all regression-based models (corrected *p* < 0.001 for all comparisons).

Bland–Altman plots illustrating the agreement between the ChatGPT-4 and SPSS-derived nomograms for sphere, cylinder, and OZ diameter are presented in Supporting Information 6. For sphere prediction, the mean difference was −0.002 with limits of agreement ranging from −0.023 to 0.019. For cylinder prediction, the mean difference was 0.001, with limits of agreement from −0.005 to 0.007. For OZ diameter, the mean difference was 0.002, with limits of agreement from −0.017 to 0.022.

To assess the consistency of AI-assisted formula generation, we additionally evaluated the results produced by ChatGPT-4o using the same dataset and regression tasks (Supporting Information 7). ChatGPT-4o selected the same variables and produced nearly identical coefficients and model structures compared to the ChatGPT-4 model (GPT-4-turbo) used in this study.

## 4. Discussion

This study demonstrates that a locally customized nomogram for SMILE surgery can be effectively developed using ChatGPT without any coding expertise. The resulting formulas performed comparably to those generated by conventional statistical software, supporting the feasibility of AI-assisted model generation in clinical settings. This approach provides a practical and accessible solution for vision correction centers aiming to optimize surgical outcomes using their own patient data. Notably, individual surgeons may prioritize different clinical variables, and the proposed method enables the creation of nomograms that reflect such preferences. While this study focused on internal validation, its primary aim was to introduce a replicable, no-code framework for personalized nomogram development.

Although the nomogram developed in this study is center-specific, the same no-code framework can be replicated by other refractive surgery centers. External users can input their own de-identified datasets into ChatGPT-4 using our published prompt structure to perform linear regression and build HTML-based calculators. This modular approach allows local retraining of the nomogram formulas without programming expertise, enabling each center to construct a customized calculator tailored to its clinical protocols and patient populations. To ensure broader applicability, future studies should include external validation using retrospective data from other institutions. A minimal validation protocol may include (1) comparing ChatGPT-predicted surgical parameters with those selected by experienced surgeons; (2) assessing refractive outcomes such as the percentage of eyes achieving postoperative spherical equivalent within ±0.50 D; and (3) stratified analysis based on patient subgroups. Additionally, multicenter prospective trials should be considered to test the model's generalizability across diverse clinical settings.

With the development of AI technologies, such as machine learning, many attempts have been made to improve the clinical results of vision correction surgery. Machine learning has been used to deeply analyze surgical safety or optimize surgical selection [15, 16]. However, algorithms developed with complex machine learning methods have not been developed because of overfitting issues and biases in measurement and surgery by institutions [17]. As ChatGPT-4 becomes popular, methods are being introduced that allow individual organizations to easily analyze their own data without the use of coding [18]. If the process conducted in this study is followed, any vision correction center can collect a small amount of data and create a customized calculator based on the analysis results, and the various functions of ChatGPT-4 can be further used to upgrade the calculator. In this study, machine learning algorithms were not employed due to their need for coding skills and specialized implementation. Instead, we demonstrated that ChatGPT-4 enables accessible and effective development of customized nomograms using linear regression. Its no-code interface allows clinicians to generate locally optimized models without technical expertise, making it a practical solution for enhancing surgical precision in vision correction procedures.

In the data recognition process, because ChatGPT is a language model, the characteristics of the variables were extracted from their names. In several experiments, it corrected errors in the dataset, such as names being entered incorrectly or pointing out outliers using its bug-fixing ability [19]. Several shortcomings were discovered during the development process. It is noteworthy that ChatGPT-4 does not always respond in the same manner to the same prompts if the command was not specific. Occasionally, ChatGPT-4 asked the user to proceed after completing an exploratory analysis of the regression analysis commands. In addition, stepwise forward or backward selection was done as the feature selection method without a specific order. This is believed to be due to ChatGPT-4's internal process, which generates various answers internally through random seeds and selects the most appropriate answer. However, we have confirmed through numerous iterative processes that the process of running the code for internal statistical calculations is carried out accurately. ChatGPT-4 clearly performed the mathematical processes accurately and presented the results. Because the intermediate calculation processes were shown in detail, it was possible to review them. The accuracy of ChatGPT-4's calculations was also confirmed in a previous study using statistical analysis using epidemiological research data [9].

Currently, the field of use of LLMs such as ChatGPT continues to expand [20]. In most medical fields, its use is focused on language-related areas such as medical information search, education, data summary, and report generation [21]. In the field of ophthalmology, it has been reported that ChatGPT can provide diagnostic assistance for ocular diseases and generate hospitalization and operative notes to assist clinicians [22, 23]. The method of using ChatGPT introduced in this study is significantly different from previous studies. This study aims to provide practical help to clinicians using the computational and programming capabilities of ChatGPT-4. In the future, with the development of more advanced LLMs, simpler software development will become possible while introducing more in-depth machine learning models to improve performance [24]. Through our research, clinicians who were unable to perform regression analysis and coding using ChatGPT-4 will be able to easily develop a nomogram calculator.

The SMILE nomogram developed in this study was also tailored to the data of a single institution, so other institutions can refer to it; however, its direct use is not recommended. We encourage clinicians to collect their own data and develop their own calculators using the proposed method. The formula developed by ChatGPT-4 required significantly fewer factors compared to previously reported machine learning nomograms [4]. Unlike the empirical nomogram [14], the previously reported nomogram receives as input not only the preoperative sphere but also all factors with high intercorrelation, such as ablation amount, residual corneal thickness, and spherical equivalent [4]. This easily causes overfitting within the machine learning algorithm, so performance degradation is expected when used in external vision correction centers. The formula developed in this study had coefficients similar to the empirical nomogram for preoperative MR, and an auxiliary adjustment of the input value according to the direction of the astigmatism axis of the MR was observed. We also found that the final formula reflected clinicians' tendency to enlarge the OZ in eyes with a wide cornea and to reduce it in cases of severe myopia to minimize lenticule depth. This was largely because of the operator's preference; therefore, the prediction power of OZ was smaller than that of the sphere and cylinder, but it was predicted in a statistically meaningful manner.

The prediction model for OZ demonstrated a relatively lower correlation coefficient (*r* = 0.491) compared to the models for sphere and cylinder, suggesting reduced predictive reliability for this parameter. This may be attributed to the multifactorial nature of OZ determination, which depends not only on biometric variables but also on subjective factors such as surgeon preference, pupil dynamics under mesopic conditions, and individualized visual demands. Despite this limitation, OZ was included in the nomogram to reflect its clinical importance in SMILE surgery planning, where consistency in OZ selection can affect postoperative visual quality and optical aberrations. Nevertheless, the lower predictive performance indicates that future studies may need to refine the modeling approach for OZ or consider decision support frameworks that incorporate both objective measurements and surgeon-specific heuristics.

Although ChatGPT was employed in this study as a supportive tool for model development and variable selection, it is essential to acknowledge the limitations of relying on AI-based language models for statistical analysis. ChatGPT is not a dedicated statistical computing platform; rather, it provides recommendations based on patterns learned from prior data. Consequently, its outputs may fail to capture dataset-specific nuances, misinterpret clinical context, or overlook key statistical assumptions. Therefore, any AI-generated guidance must be critically assessed and validated using appropriate statistical software. While ChatGPT can enhance accessibility for clinicians without programming expertise, its use should be grounded in domain expertise and complemented by rigorous statistical verification to ensure methodological robustness and clinical reliability. Regarding privacy, all patient data were anonymized and processed locally prior to use. Since ChatGPT operates in a conversational prompt format, no patient identifiers were uploaded. In practical deployment, we recommend that the HTML-based calculator includes a safeguard requiring manual review and confirmation by the surgeon before finalizing surgical parameters.

Compared to commercial planning tools such as Zeiss Refractive Workplace, our ChatGPT-based approach is more cost-effective and flexible. Users can freely add or remove variables to develop customized models tailored to local protocols and patient characteristics. In contrast, commercial software typically relies on fixed inputs within closed systems. Our method also ensures transparency, allowing clinicians to inspect and modify both the regression formulas and interface design. While not a substitute for regulatory-approved tools, it offers an accessible and adaptable alternative for clinician-led nomogram development.

This study had several limitations. First, we did not compare Gemini, Claude3, or other recently released LLMs separately. They have shown similar performance to ChatGPT-4 [25]. Currently, ChatGPT-4 is becoming the standard for LLMs, so comparison may be necessary in the future. Second, this was a single-center study limited to East Asian populations. The cornea and refractive power of Caucasians may have different anatomical and optical characteristics, so caution is required for external application [26]. The primary objective of this study was not external validation, but rather to demonstrate the feasibility of developing a locally optimized nomogram tailored to the specific patient demographics and surgical practices of a single center. Third, this study was a retrospective analysis. Additional research is needed to determine whether clinical outcomes and clinician satisfaction will improve prospectively by using the developed calculator. Fourth, there was a statistically significant difference in preoperative cylinder values between the training and validation sets, which may have introduced bias in evaluating the model's performance for cylindrical correction. Although the overall predictive accuracy remained stable, future studies should consider stratified sampling to ensure more balanced distributions of key refractive parameters.

## 5. Conclusion

Locally tailored nomograms are essential in refractive surgery due to variations in surgical techniques, patient demographics, and diagnostic equipment across institutions. This study demonstrated a practical, no-code approach to building customized nomograms using ChatGPT-4, which enabled linear regression model development with feature selection and facilitated the generation of a web-based calculator. The resulting models showed superior predictive performance for postoperative refraction compared to a simple empirical nomogram. Importantly, this workflow allows clinicians to independently create accurate, institution-specific nomograms using their own datasets without programming expertise. While external validation was not the primary aim, the approach was intended to empower individual centers to develop their own predictive tools. This study establishes a foundational method for accessible, AI-assisted nomogram development in clinical practice.

## Figures and Tables

**Table 1:** ChatGPT-4 prompts used in this study.

Task	Detailed prompt
1. Load the dataset	(After dragging the dataset) This is a dataset to develop a nomogram for SMILE (small-incision lenticule extraction) surgery

2. Develop nomogram formulas to calculate the parameters for VISUMAX 500	Create a formula to predict “Parameter_SPH” using a linear regression analysis. Perform feature selection starting with the following variables as dependent variables
	MR_sph, MR_cyl, MR_axis, WTW, CCT, Pupil_size, K_mean, K_astig
	Create a formula to predict “Parameter_CYL” using a linear regression analysis. Perform feature selection starting with the following variables as dependent variables
	MR_sph, MR_cyl, MR_axis, WTW, CCT, Pupil_size, K_mean, K_astig
	Create a formula to predict “Parameter_OZ” using a linear regression analysis. Perform feature selection starting with the following variables as dependent variables
	MR_sph, MR_cyl, MR_axis, WTW, CCT, Pupil_size, K_mean, K_astig

3. Organize formulas and input data	What are the variables needed to calculate the three formulas Parameter_SPH, Parameter_CYL, and Parameter_OZ above?

4. Create HTML codes to build a nomogram calculator	Create a nomogram calculator that calculates Parameter_SPH, Parameter_CYL, and Parameter_OZ values using above formulas
	Build the codes for the nomogram calculator written in html, css, and javascript in one html file
	Design
	The text boxes must have rounded edges
	Make this calculator look professional by creating a frame around it
	Separate the title, input window, and output window frames
	Insert the title of the calculator in the title frame above the input window frame. The title is “SMILE (ReLE for VISUMAX 500) nomogram calculator”
	Add the subtitle of the input window inside the frame: “Preoperative examinations”
	Add the subtitle of the output window inside the frame: “Parameters for VISUMAX 500”
	The calculator should have a maximum width of 500 pixels
	The input frame should be above, and the output frame should be below. The title, input, and output frames must be aligned in order from the top
	Place the calculator in the center of the screen

5. Additionally design the calculator	Sort the input items into two columns
	In the “MR_sph,” “MR_cyl,” “K_mean,” and “K_astig” input sections, add the unit: (D)
	In the “Pupil_size” and “WTW” input sections, add the unit: (mm)
	In the “MR_axis” input section, add a supplementary explanation: “Against-the-rule: 1/With-the-rule: 0”
	Please arrange the calculation results so that they are on one line with the description of each item.
	In the “Parameter_SPH” and “Parameter_CYL” output sections, add the unit: (D)
	In the “Parameter_OZ” output sections, add the unit: (mm)

6. Set initial values	Select randomly one case from the dataset and set its variables as initial input values

**Table 2:** Clinical measurements of study participants.

	Training set (*N* = 950 eyes)	Test set (*N* = 318)	*p* value
*Preoperative data*			
MR sphere (D)	−3.64 ± 1.28	−3.78 ± 1.32	0.094
MR cylinder (D)	−0.84 ± 0.65	−0.80 ± 0.59	0.319
MR axis (against-the-rule, %)	89 (9.4)	30 (9.4)	0.999
White-to-white diameter (mm)	11.73 ± 0.38	11.72 ± 0.39	0.917
Central corneal thickness (μm)	552.44 ± 27.28	555.51 ± 29.05	0.087
Pupil size (mm)	3.01 ± 0.55	3.05 ± 0.57	0.258
Keratometry mean (D)	43.00 ± 1.31	42.98 ± 1.32	0.776
Keratometry astigmatism (D)	1.40 ± 0.65	1.39 ± 6.25	0.808

*Postoperative data at 6 months*			
MR sphere (D)	0.23 ± 0.24	0.21 ± 0.23	0.073
MR cylinder (D)	−0.18 ± 0.18	−0.21 ± 0.18	0.001

*VISUMAX 500 input parameters*			
Sphere (D)	−4.12 ± 1.41	−4.23 ± 1.46	0.217
Cylinder (D)	−0.96 ± 0.67	−0.93 ± 0.61	0.428
Optical zone diameter (mm)	6.33 ± 0.14	6.30 ± 0.15	0.001

*Note:* D, diopters.

Abbreviation: MR, manifest refraction.

**Table 3:** Results of linear regression with stepwise backward elimination performed by SPSS to calculate VISUMAX 500 input parameters.

Target variable (VISUMAX 500 input parameters)	Included input variable	Coefficient (B)	*p* value
Sphere (Parameter_SPH, D)	Constant	−0.140	< 0.001
	MR sphere (MR_sph)	1.095	< 0.001
	MR cylinder (MR_cyl)	0.023	0.041
	Keratometry astigmatism (K_astig)	0.019	0.096

Cylinder (Parameter_CYL, D)	Constant	−0.075	< 0.001
	MR sphere (MR_sph)	−0.005	0.076
	MR cylinder (MR_cyl)	0.903	< 0.001
	MR axis (MR_axis, against-the-rule)	0.156	< 0.001
	Keratometry astigmatism (K_astig)	−0.117	< 0.001

Optical zone diameter (Parameter_OZ, mm)	Constant	4.257	< 0.001
	MR sphere (MR_sph)	0.027	< 0.001
	White-to-white diameter (WTW)	0.168	< 0.001
	Central corneal thickness (CCT)	3.65 ∗ 10^−4^	0.012

**Table 4:** Summary of prediction error metrics for each nomogram model.

Type	Regression tool	MAE	MedAE	RMSE
Sphere (D)	ChatGPT-4	0.3081	0.2487	0.3795
	R	0.3093	0.2568	0.3808
	SPSS	0.3077	0.2513	0.3782
	Simple nomogram	0.4349	0.4141	0.5076

Cylinder (D)	ChatGPT-4	0.2524	0.2206	0.3174
	R	0.2526	0.223	0.3176
	SPSS	0.2515	0.2195	0.3163
	Simple nomogram	0.1817	0.1625	0.2388

Spherical equivalent (D)	ChatGPT-4	0.2696	0.2349	0.3284
	R	0.2691	0.2443	0.3283
	SPSS	0.2699	0.2405	0.3282
	Simple nomogram	0.4352	0.4441	0.5104

*Note:* The table presents mean absolute error (MAE), median absolute error (MedAE), and root mean square error (RMSE) in diopters (D) for each model: ChatGPT-4-based, R-based, SPSS-based, and the Simple nomogram. Lower values indicate better predictive accuracy and robustness. Lower values indicate higher predictive accuracy and consistency.

## Data Availability

The prompts used in this study are shown in the Supporting Information. The datasets utilized during this study are not publicly available. Data are available upon reasonable request.

## Nomenclature

CCT: Central corneal thickness
HTML: Hypertext Markup Language
MR_axis: Axis of manifest astigmatism
MR_cyl: Preoperative cylinder
MR_sph: Preoperative sphere
SMILE: Small-incision lenticule extraction
WTW: White-to-white corneal diameter

## Supporting Information

Supporting Information 1. The dataset structure.

Supporting Information 2. R code for linear regression model development, stepwise optimization, and validation.

Supporting Information 3. Feature selection and linear regression processes performed by ChatGPT-4.

Supporting Information 4. The calculator codes generated by ChatGPT-4 to develop a laser vision correction (SMILE) nomogram.

Supporting Information 5. Screenshot of R output showing linear regression models used to predict nomogram parameters for SMILE.

Supporting Information 6. Bland–Altman plots to compare ChatGPT-4 and SPSS.
