# Supplementary material for: Codeless Development of a Customized SMILE Nomogram Using a Large Language Model: A Practical Framework for Clinicians
Source: J Ophthalmol. 2025 Jul 15;2025:9930116. doi: 10.1155/joph/9930116 (PMC12283199; doi:10.1155/joph/9930116)
Supplement: Supporting Information — Additional supporting information can be found online in the Supporting Information section. [file 9930116.f1.pdf]

Supplementary Materials

Supplementary Material 1. The dataset structure.

Example of data set - Column names used in this study were presented.

	A	B	C	D	E	F	G	H	I	J	K
1	MR_sph	MR_cyl	MR_axis	WTW	CCT	Pupil_size	K_mean	K_astig	Parameter_SPH	Parameter_CYL	Parameter_OZ
2	-3.75	-0.25	1	11.7	591	3.03	43.7	1	-4.35	-0.25	6.3
3	-3.75	0	0	11.7	587	3.16	44	1.2	-4.35	-0.2	6.3
4	-3	0	0	11.4	512	3.92	44.2	1	-3.25	-0.1	6.3
5	-2.25	0	0	11.4	506	3.9	44.6	1.2	-2.5	-0.2	6.3
6	-2.75	-0.5	0	11.4	541	2.55	43.3	0.6	-3.1	-0.7	6.4
7	-3.75	-0.25	0	11.4	533	2.85	43.5	0.7	-4.3	-0.4	6.4
8	-2.25	-0.5	1	11.5	568	3.4	43.1	0.5	-2.7	-0.5	6.3
9	-2.25	-0.25	1	11.5	572	3.12	43.2	0.9	-2.7	-0.25	6.3
10	-5.12	-0.25	0	11.9	552	3.4	41.8	1	-5.78	-0.35	6.3
11	-5.37	-0.5	0	11.9	551	3	41.9	1	-6.05	-0.6	6.3
12	-2.75	-2	0	11.5	551	2.78	44.5	2.7	-3.1	-2.1	6.5
13	-1.5	-0.25	0	12.1	570	2.87	42.5	1.1	-1.75	-0.35	6.3
14	-1.75	0	0	12.1	576	2.93	42.7	1.1	-2.1	-0.2	6.3
15	-2.5	0	0	11	543	3.66	44.5	0.2	-2.75	0	6.1
16	-4.25	-0.75	0	12.5	545	2.82	40.1	0.9	-4.7	-0.75	6.4
17	-4.37	-0.5	0	12.5	531	3.06	40.9	1.1	-4.8	-0.75	6.4
18	-3.87	-0.75	1	12.6	535	2.77	40.7	0.3	-4.25	-0.6	6.5
19	-4.12	-0.25	1	12.5	536	2.83	40.9	0.3	-4.8	-0.1	6.5

CCT, central corneal thickness (um)

K_mean, mean keratometry of corneal anterior surface (D)

K_astig, keratometry astigmatism (difference between steep and flat) of corneal anterior surface (D)

MR_axis, axis of manifest astigmatism, between 45° and 135° and was coded as 1 for against-the-rule astigmatism and 0 for with-the-rule astigmatism

MR_cyl, preoperative cylinder (D)

MR_sph, preoperative sphere (D)

WTW, white-to-white corneal diameter (mm)

Supplementary Material 2. R code for linear regression model development, stepwise optimization, and validation.

```
install.packages("readxl")     # For reading Excel files
install.packages("MASS")       # For stepwise regression
install.packages("Metrics")    # For MAE and RMSE

# Load required libraries
library(readxl)
library(MASS)    # For stepAIC
library(Metrics) # For MAE and RMSE

# Load training and validation datasets
train <- read_excel("data_train.xlsx")
val <- read_excel("data_validation.xlsx")

# Define formula
full_formula <- as.formula("~ MR_sph + MR_cyl + MR_axis + WTW + CCT + Pupil_size + K_mean
+ K_astig")

# Function to build model, predict, and evaluate
evaluate_model <- function(target, train, val) {
  # Build formula
  fml <- as.formula(paste(target, "~ ."))

  # Subset training and validation data
  predictors <- all.vars(full_formula)
  train_sub <- train[, c(target, predictors)]
  val_sub <- val[, predictors]
  y_true <- val[[target]]

  # Fit full model and perform stepwise AIC
  full_model <- lm(fml, data = train_sub)
  step_model <- stepAIC(full_model, direction = "both", trace = FALSE)
  print(summary(step_model))

  # Predict on validation
  y_pred <- predict(step_model, newdata = val_sub)

  # Evaluate
  mae_val <- mae(y_true, y_pred)
  rmse_val <- rmse(y_true, y_pred)
  cat(paste0("Performance for ", target, ":\n"))
  cat(paste0("  MAE  = ", round(mae_val, 3), "\n"))
  cat(paste0("  RMSE = ", round(rmse_val, 3), "\n\n"))

  return(list(model = step_model, prediction = y_pred))
}

# Run for each target variable
model_sph <- evaluate_model("Parameter_SPH", train, val)
model_cyl <- evaluate_model("Parameter_CYL", train, val)
model_oz  <- evaluate_model("Parameter_OZ", train, val)
```

Supplementary Material 3. Feature selection and linear regression processes performed by ChatGPT-4.

It is noteworthy that ChatGPT-4 does not always respond in the same manner to the same prompts. Occasionally, ChatGPT-4 asked the user to proceed after completing an exploratory analysis of the regression analysis commands.
This process was done using random sampled data set for example purposes, so there are some differences from the result formula in the main text.

T **You**

Create a formula to predict "Parameter_SPH" using a linear regression analysis. Perform feature selection starting with the following variables as dependent variables.

MR_sph, MR_cyl, MR_axis, WTW, CCT, Pupil_size, K_mean, K_astig

ChatGPT

To start, I'll load your dataset to understand its structure, and then we'll proceed with feature selection and building a linear regression model to predict "Parameter_SPH". Let's first load and examine your data.

The dataset is loaded, and it includes several variables that we can use for predicting "Parameter_SPH". Here's a brief summary of the dataset:

- **MR_sph**: Spherical measurement from manifest refraction.
- **MR_cyl**: Cylindrical measurement from manifest refraction.
- **MR_axis**: Axis measurement from manifest refraction.
- **WTW**: White-to-white distance.
- **CCT**: Central corneal thickness.
- **Pupil_size**: Diameter of the pupil.
- **K_mean**: Mean keratometry.
- **K_astig**: Keratometric astigmatism.
- **Parameter_SPH**: The outcome variable we want to predict.

Next, I'll proceed with feature selection and then build a linear regression model using these variables to predict "Parameter_SPH". We'll start by using correlation analysis to select features that have a strong relationship with "Parameter_SPH". Then, we'll fit a linear regression model using those selected features. Let's start with the feature selection process.

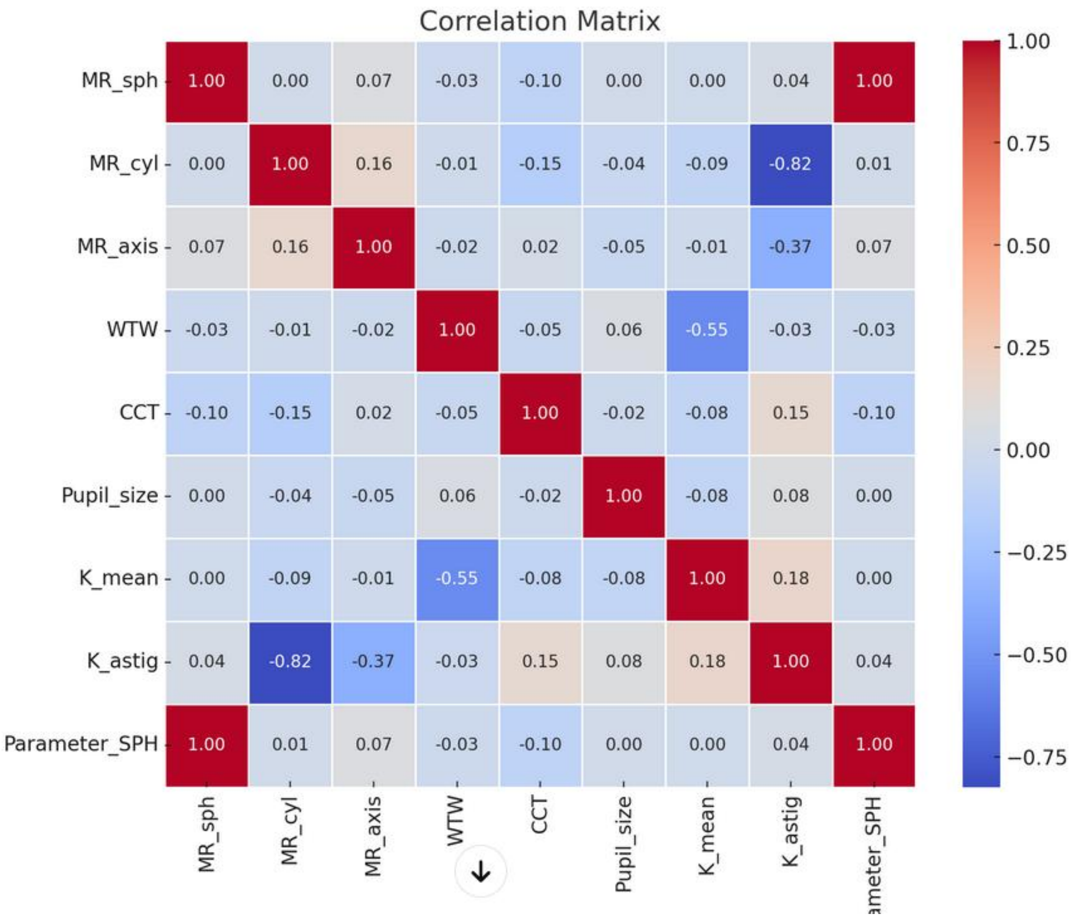

The correlation matrix provides insights into how different variables relate to "Parameter_SPH". Here are some observations:

- **MR_sph** shows a strong positive correlation with "Parameter_SPH", which suggests it's a significant predictor.
- Other variables like **MR_cyl**, **WTW**, **CCT**, **Pupil_size**, **K_mean**, and **K_astig** have varying degrees of correlation.

Given the strong correlation of **MR_sph** and some contribution from other features, I will proceed to create a linear regression model starting with all these features, and then refine the model by removing features that don't significantly contribute to the prediction.

I'll begin by fitting the initial model and then examine the statistical significance of each feature to decide on their inclusion. Let's proceed with building the linear regression model.
The initial regression model for predicting "Parameter_SPH" has a very high R^2 value of 0.991, indicating that our model explains nearly 99.1% of the variance in "Parameter_SPH". However, some of the variables might not be significantly contributing to the model. Here's a brief overview of the results:

Model Summary

- **MR_sph**: Highly significant with a strong coefficient (1.0958), indicating a direct and substantial influence on "Parameter_SPH".
- **MR_cyl**, **K_astig**: These have some significance but their coefficients are quite small.
- **MR_axis**, **WTW**, **CCT**, **Pupil_size**, **K_mean**: These variables are not statistically significant ($p > 0.05$) and have very small coefficients.

The simplified linear regression model provides a concise formula for predicting "Parameter_SPH" using only "MR_sph", "MR_cyl", and "K_astig". The model remains highly effective, with an R^2 value of 0.992, indicating it explains about 99.2% of the variance in "Parameter_SPH".

Simplified Model Formula

$$\text{Parameter\_SPH} = -0.1404 + 1.0954 \times \text{MR\_sph} + 0.0233 \times \text{MR\_cyl} + 0.0191 \times \text{K\_astig}$$

Coefficients and Significance

- **Intercept**: -0.1404
- **MR_sph**: Coefficient = 1.0954, highly significant ($p < 0.001$), indicating a strong positive influence on "Parameter_SPH".
- **MR_cyl**: Coefficient = 0.0233, marginally significant ($p = 0.041$).
- **K_astig**: Coefficient = 0.0191, not statistically significant ($p = 0.096$), but might still offer some clinical relevance.

This model is more manageable and focuses on the most influential predictors. If you have any further analysis or specific tasks in mind, feel free to let me know! [>-]

Supplementary Material 4. The calculator codes generated by ChatGPT-4 to develop a laser vision correction (SMILE) nomogram.

```
<!DOCTYPE html>
<html lang="en">
<head>
<meta charset="UTF-8">
<title>SMILE (ReLE for VISUMAX 500) Nomogram Calculator</title>
<style>
    body {
        font-family: Arial, sans-serif;
        display: flex;
        justify-content: center;
        align-items: center;
        height: 100vh;
        margin: 0;
        background-color: #f4f4f4;
    }
    .container {
        max-width: 500px;
        width: 100%;
        text-align: center;
    }
    .frame {
        border: 2px solid #007BFF;
        padding: 20px;
        margin-top: 20px;
        background-color: #fff;
        border-radius: 10px;
    }
    .title, .subtitle {
        text-align: center;
        font-size: 18px;
        font-weight: bold;
        margin-bottom: 10px;
    }
    .subtitle {
        font-size: 16px;
        color: #333;
        margin-bottom: 10px;
    }
    .inputs {
        display: flex;
        flex-wrap: wrap;
        justify-content: space-between;
    }
    .input-group {
        width: 48%; /* Adjust width for two columns */
```

```
        margin-bottom: 10px;
    }
    label {
        display: block;
        margin-bottom: 5px;
    }
    input, output {
        width: 100%;
        padding: 8px;
        margin-top: 5px;
        border: 1px solid #ccc;
        border-radius: 8px;
    }
    button {
        padding: 10px 20px;
        margin-top: 20px;
        width: 100%;
        background-color: #007bff;
        color: white;
        border: none;
        border-radius: 8px;
        cursor: pointer;
    }
    button:hover {
        background-color: #0056b3;
    }
    .note {
        font-size: 12px;
        color: #888;
        margin-top: 5px;
    }
</style>
<script>
    function calculateValues() {
        var mr_sph = parseFloat(document.getElementById('mr_sph').value);
        var mr_cyl = parseFloat(document.getElementById('mr_cyl').value);
        var mr_axis = parseFloat(document.getElementById('mr_axis').value);
        var pupil_size = parseFloat(document.getElementById('pupil_size').value);
        var k_astig = parseFloat(document.getElementById('k_astig').value);
        var wtw = parseFloat(document.getElementById('wtw').value);

        var parameter_sph = (-0.1200 + 1.0983 * mr_sph - 0.0284 * mr_axis).toFixed(4);
        var parameter_cyl = (-0.0934 - 0.0062 * mr_sph + 0.9043 * mr_cyl + 0.1577 * mr_axis + 0.0032 *
pupil_size - 0.1125 * k_astig).toFixed(4);
        var parameter_oz = (4.4964 + 0.0257 * mr_sph + 0.1646 * wtw).toFixed(4);

        document.getElementById('result_sph').value = parameter_sph;
        document.getElementById('result_cyl').value = parameter_cyl;
```

```
        document.getElementById('result_oz').value = parameter_oz;
    }
</script>
</head>
<body>
    <div class="container">
        <div class="title">SMILE (ReLE for VISUMAX 500) nomogram calculator</div>
        <div class="frame">
            <div class="subtitle">Preoperative examinations</div>
            <div class="inputs">
                <div class="input-group">
                    <label for="mr_sph">MR_sph (D):</label>
                    <input id="mr_sph" type="number" step="any">
                    <div class="note">Range: -7.0 to -0.5</div>
                </div>
                <div class="input-group">
                    <label for="mr_cyl">MR_cyl (D):</label>
                    <input id="mr_cyl" type="number" step="any">
                    <div class="note">Range: -3.0 to 0.0</div>
                </div>
                <div class="input-group">
                    <label for="mr_axis">MR_axis (0 or 1):</label>
                    <input id="mr_axis" type="number" step="any">
                    <div class="note">Against-the-rule: 1 / With-the-rule: 0</div>
                </div>
                <div class="input-group">
                    <label for="pupil_size">Pupil_size (mm):</label>
                    <input id="pupil_size" type="number" step="any">
                    <div class="note">Range: 1.6 to 5.7</div>
                </div>
                <div class="input-group">
                    <label for="k_astig">K_astig (D):</label>
                    <input id="k_astig" type="number" step="any">
                    <div class="note">Range: 0.0 to 3.4</div>
                </div>
                <div class="input-group">
                    <label for="wtw">WTW (mm):</label>
                    <input id="wtw" type="number" step="any">
                    <div class="note">Range: 10.7 to 13.0</div>
                </div>
            </div>
            <button onclick="calculateValues()">Calculate</button>
        </div>
        <div class="frame">
            <div class="subtitle">Parameters for VISUMAX 500</div>
            <label for="result_sph">Parameter_SPH:</label>
            <output id="result_sph"></output>
            <label for="result_cyl">Parameter_CYL:</label>
```

```html
            <output id="result_cyl"></output>
            <label for="result_oz">Parameter_OZ:</label>
            <output id="result_oz"></output>
        </div>
    </div>
</body>
</html>
```

Supplementary Material 5. Screenshot of R output showing linear regression models used to predict nomogram parameters for SMILE.

```
> model_sph <- evaluate_model("Parameter_SPH", train, val)

Call:
lm(formula = Parameter_SPH ~ MR_sph + MR_cyl + K_astig, data = train_sub)

Residuals:
     Min       1Q   Median       3Q      Max 
-0.61187 -0.07539 -0.01117  0.06901  0.48883 

Coefficients:
             Estimate Std. Error t value Pr(>|t|)    
(Intercept) -0.140378   0.016419  -8.550   <2e-16 ***
MR_sph       1.095405   0.003292 332.759   <2e-16 ***
MR_cyl       0.023296   0.011387   2.046   0.0410 *  
K_astig      0.019057   0.011422   1.669   0.0955 .  
---
Signif. codes:  0 '***' 0.001 '**' 0.01 '*' 0.05 '.' 0.1 ' ' 1

Residual standard error: 0.1301 on 946 degrees of freedom
Multiple R-squared:  0.9916,	Adjusted R-squared:  0.9916 
F-statistic: 3.713e+04 on 3 and 946 DF,  p-value: < 2.2e-16

> model_cyl <- evaluate_model("Parameter_CYL", train, val)

Call:
lm(formula = Parameter_CYL ~ MR_sph + MR_cyl + MR_axis + K_astig, 
    data = train_sub)

Residuals:
     Min       1Q   Median       3Q      Max 
-1.10380 -0.07279  0.00999  0.07357  0.34505 

Coefficients:
             Estimate Std. Error t value Pr(>|t|)    
(Intercept) -0.074780   0.015221  -4.913 1.06e-06 ***
MR_sph      -0.004939   0.002779  -1.777   0.0758 .  
MR_cyl       0.903422   0.009943  90.861  < 2e-16 ***
MR_axis      0.155935   0.013676  11.402  < 2e-16 ***
K_astig     -0.116788   0.010611 -11.006  < 2e-16 ***
---
Signif. codes:  0 '***' 0.001 '**' 0.01 '*' 0.05 '.' 0.1 ' ' 1

Residual standard error: 0.1091 on 945 degrees of freedom
Multiple R-squared:  0.9739,	Adjusted R-squared:  0.9738 
F-statistic:  8826 on 4 and 945 DF,  p-value: < 2.2e-16

> model_oz  <- evaluate_model("Parameter_OZ", train, val)

Call:
lm(formula = Parameter_OZ ~ MR_sph + WTW + CCT, data = train_sub)

Residuals:
     Min       1Q   Median       3Q      Max 
-0.46542 -0.07644 -0.01956  0.07003  0.42315 

Coefficients:
             Estimate Std. Error t value Pr(>|t|)    
(Intercept) 4.2565989  0.1487150  28.623   <2e-16 ***
MR_sph      0.0266657  0.0030744   8.673   <2e-16 ***
WTW         0.1682036  0.0103634  16.231   <2e-16 ***
CCT         0.0003650  0.0001451   2.515   0.0121 *  
---
Signif. codes:  0 '***' 0.001 '**' 0.01 '*' 0.05 '.' 0.1 ' ' 1

Residual standard error: 0.1212 on 946 degrees of freedom
Multiple R-squared:  0.2591,	Adjusted R-squared:  0.2568 
F-statistic: 110.3 on 3 and 946 DF,  p-value: < 2.2e-16
```

Supplementary Material 6. Bland-Altman plots to compare ChatGPT-4 and SPSS

(A) SPH

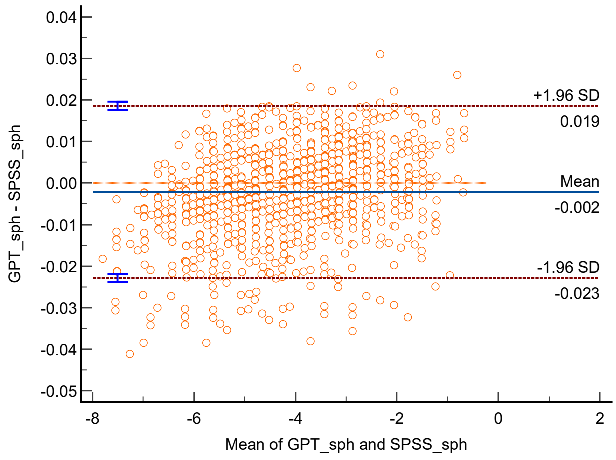

(B) CYL

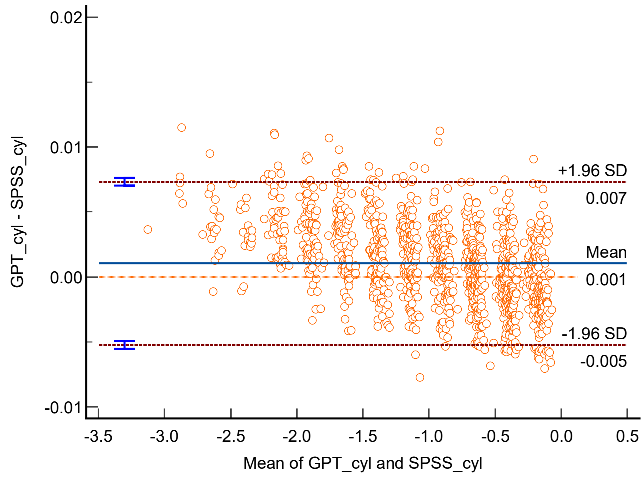

(C) Optic zone size

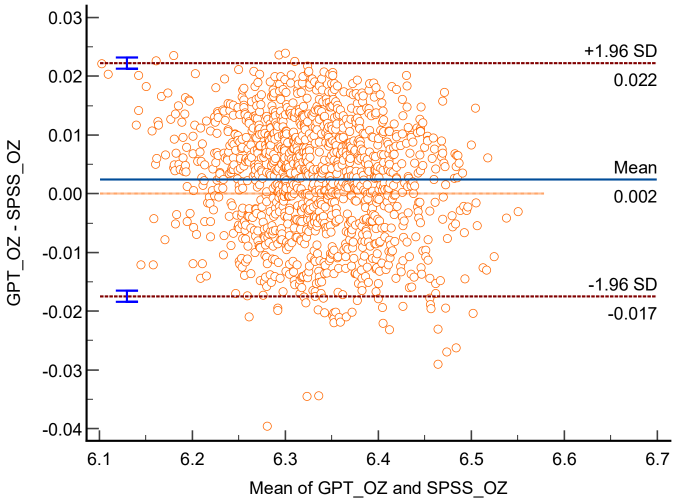

Supplementary Material 7. Regression outputs generated by ChatGPT-4o using the same input dataset and modeling instructions as those applied with ChatGPT-4 (GPT-4-turbo). The selected variables, regression coefficients, and model structures for both Parameter_SPH and Parameter_CYL closely match those obtained using GPT-4-turbo, demonstrating consistency and reproducibility across AI model versions.

Create a formula to predict "Parameter_SPH" using a linear regression analysis. Perform feature selection starting with the following variables as dependent variables.

MR_sph, MR_cyl, MR_axis, WTW, CCT, Pupil_size, K_mean, K_astig

The linear regression analysis to predict **Parameter_SPH** yields the following results:

Variable	Coefficient	p-value
**MR_sph**	**+1.0958**	<0.001
MR_axis	−0.0259	0.063
Intercept	−0.1196	<0.001

🔍 Interpretation

- `MR_sph` **remains highly significant** and dominates the model.
- `MR_axis` **has a borderline p-value (0.063)**, suggesting limited or questionable contribution.
- Removing `MR_axis` does **not materially reduce R²** (still **0.990**).

Formula:

$$\text{Parameter\_SPH} = -0.1196 + 1.0958 \cdot \text{MR\_sph} - 0.0259 \cdot \text{MR\_axis}$$

Create a formula to predict "Parameter_CYL" using a linear regression analysis. Perform feature selection starting with the following variables as dependent variables.

MR_sph, MR_cyl, MR_axis, WTW, CCT, Pupil_size, K_mean, K_astig

✅ Final Linear Regression Model for Predicting `Parameter_CYL`

Variable	Coefficient	p-value
**MR_cyl**	**+0.9018**	<0.001
**MR_axis**	**+0.1532**	<0.001
**K_astig**	**−0.1189**	<0.001
**Intercept**	−0.0549	<0.001

> `MR_sph` and `Pupil_size` were eliminated due to insignificance.

Model Performance:

- **R² = 0.974**
- **Adjusted R² = 0.974**
- Excellent predictive power and parsimony

Final Formula:

$$\text{Parameter\_CYL} = -0.0549 + 0.9018 \cdot \text{MR\_cyl} + 0.1532 \cdot \text{MR\_axis} - 0.1189 \cdot \text{K\_astig}$$
